# Modern Sociology

**Published:** 1899-03-25

**Authors:** 


### Modern Sociology.

### THE MODEL LODGING HOUSE FOR WIDOWS AND WIDOWERS AND THEIR CHILDREN IN GLASGOW.

It is no new thing with us to look to Glasgow for precedents. It would take some time to enumerate the many practical experiments in economics and sociology which have been carried on under the auspices of Glasgow merchants and bailies. It is interesting, too, to note what Mr. Bryce, as president of the Glasgow Civic Society, pointed out last winter in his inaugural address, that Durban, under its Scotch mayor, is attempting to do for our colonial towns in Africa what Glasgow is doing for our home municipalities—setting an example, by putting into practice, often at great cost, the theories of civic science.

One great problem which faces all students of practical economics is that of widows and widowers, who, having young children, are unable to look after them themselves, and who have no relative who can undertake to do this for them. The impossibility of keeping such children under proper control, and the want of adequate care for their personal well-being, seems to be self-evident. It was proposed to build a model family lodging house for widows and widowers and their families, and this has been done in the centre of Glasgow, near the site of the old University, which has been replaced by the goods station.

The Family Lodging House is not without architectural merit. It contains accommodation for some 300 persons. Its wide corridors, painted in chocolate-red, its large windows on the landings, &c., compare favourably with some halls of residence for women students in which the inmates pay many times over the sum required here. Each inhabitant has a comfortable, neat, and nicely-kept bedroom, and there are large recreation-rooms on each landing, the common property of men and women. These rooms are large and well built, they have many windows, and are simply furnished in American cane. The central tables are supplied with papers and journals, so that the house partakes of the nature of a club.

Judging by the accommodation allotted to them, here, too, the children may be said to "boss the show." They have several recreation-rooms for games, &c., and an asphalte outdoor playground, provided with wire nets to keep in eccentric balls. Their refectory is very well managed. They are allowed excellent porridge, with unlimited fresh milk, for breakfast, and those very un-national children who do not like porridge are allowed tea and bread and jam.

All meals are taken under the personal supervision of the matron, who is a mother to these poor little orphans, and who insists on their eating a fair supply. We may note in passing that the kitchen machinery is on a huge scale, and repays a visit.

Ample provision is made in this house for that "cleanliness which is next to goodliness," and some of the latest improvements, scarcely to be met with outside a hydropathic establishment, are to be seen here. Patent sprays for warm and cold water, metal walls to keep out draughts of cold air, are all lavished on these fortunate young people—we wonder how many of them go in for voluntary shower baths! No doubt it is some little time before new comers feel friendly towards these novelties.

There is no doubt that the children thrive under the kindly *régime* of the matron. Their physical needs are considered; good food, cleanliness, proper recreation, and inducements to sound sleep are all thoroughly provided here; and, as everyone knows, the instruction provided by the Scotch schools is of a very high order. These children may then be considered as under a special dispensation, and have so many opportunities that they can, we would think, hardly do less than reward the originators of the scheme by turning out as efficient members of the community, ready equipped for the battle of life, and prepared in their turn to act up to a liberal standard of citizenship.

But while the children are gaining by living under these highly civilising surroundings, the institution is less popular with the fathers and mothers than we might have been led to expect. They seem to feel a certain undefinable constraint which was not reckoned for by the promoters of the scheme. There is a certain shyness about meeting in the sitting-rooms, a lack of conversation which is apt to turn to silence, and a tendency among the men to spend their leisure and summer evenings on the comfortable settees provided in the window niches on the landings, enjoying a friendly pipe. Certain it is that the building is not yet full, which seems such a mistake when one thinks of the acres of wretche[illegible] lodgings all around; for, be remembered, the institution, though itself on a tram thoroughfare, is at the same time in the midst of the slums. If not as yet an absolute success the scheme is very far from being a failure; and when we consider the extreme independence of the Scottish character—"the touch-me-not" respectability which is the aim of so many, the hard surroundings in which the candidates for quarters have been themselves brought up—we wonder rather at the possibility of putting such a sociable scheme into such efficient working order. If one thing strikes a Southerner more than another when making a stay in Scotch towns it is the peculiarities of a Scotch crowd. There is no happy chatting of groups interchanging friendly or neighbourly greetings as in France, but a standing about of hundreds of men absorbed on some more or less evident problem, ignoring alike themselves, their neighbours, and even the rain; while the few women who are not chilled off by this exclusive view of things stand apart by themselves with shawled heads. These people are incapable of understanding the need of amusement; they live and die in harness, and have no time to play. For personalities such as these the home is the pivot round which the universe turns. Community life, as such, cannot appeal to the Scotch. One can understand that a pipe at a landing window may, after all, be the sweetest compensation for the loss of all that which we call "home."

